# Laparoscopic total hysterectomy still not routinely chosen Operative description and available instruments

**DOI:** 10.25122/jml-2019-0051

**Published:** 2019

**Authors:** Alexandra Popa, Catalin Copaescu, Valerica Horhoianu

**Affiliations:** 1. Obstetrics & Gynecology Department, Emergency University Hospital, Bucharest, Romania; 2. General Surgery Department, Ponderas Hospital, Bucharest, Romania

**Keywords:** total hysterectomy, laparoscopy, minimally invasive surgery

## Abstract

Hysterectomy is the most common gynecological surgical intervention; therefore, there are many technical variations in different healthcare systems around the world. We aimed to review, step by step, the technique of laparoscopic hysterectomy as well as to present the available variety of surgical instruments impartially so that the operative team can decide in an informed manner the model and characteristics of the equipment used. The surgical technique is presented based on the experience of the authors, focusing mainly on intraoperative recommendation and suggestions. Advantages and disadvantages of the available instruments are also extensively detailed. Surgical positioning, as well as inserting the uterine manipulator are essential steps. The open technique is used to create pneumoperitoneum. The utero-ovarian ligament or the infundibulopelvic ligament is identified, coagulated and cut. The round ligament is incised, entering the space between the two layers of the broad ligament and advancing caudally in this space, which, if correctly identified, should be avascular. The uterine vessels located on the posterior sheet of the broad ligament are dissected and coagulated. The vaginal wall is sectioned with the help of the manipulator’s cap, making it easier to expose the insertion line of the vagina on the cervix. The uterus is removed through the vagina or through a trans-parietal incision. Thereafter, the vagina is sutured using separate Vicryl sutures. Between 2011 and 2016, laparoscopic hysterectomy had an increasing trend all over Europe. With a reported percentage of 3%, Romania ranks last in hysterectomies performed laparoscopically. The laparoscopic approach offers the advantages of minimal invasiveness: less pain, faster recovery and early social reintegration; therefore, this trend of improvement should become more accepted.

## Introduction

Hysterectomy is the most common gynecological surgical intervention, and there are evident technical variations regarding the procedure in different healthcare systems around the world. Although there is a tendency to decrease the number of hysterectomies in western countries due to the extension of the conservative medical approach, it remains a procedure with a particularly important impact, both on the economy and quality of life of a large segment of the female population. Currently, hysterectomy is the eighth most frequent intervention performed in the healthcare systems in Europe. In terms of incidence, figures are reported in the literature varying from 5.4 hysterectomies/1000 women in the USA to 3.7/1000 in Italy, 1.8/1000 in Denmark and 2.1/1000 in Sweden [1-4]. It is considered that the rate of hysterectomies relative to the number of females has decreased by about 1 ‰ per decade since the 1980s. Even considering these epidemiological aspects, it is estimated that approximately 20% of women will be hysterectomized before 70 years of age [5-8].

Regarding the surgical approach, the Statistical Office of The European Communities (Eurostat) reported in 2016 that more than 50% of all hysterectomies performed in Finland, Poland, the Czech Republic, Slovakia, Estonia and Belgium were performed laparoscopically, while lower rates of 33-40% for this approach were recorded in the Netherlands, Austria, France, Lithuania and Germany. In the rest of the European countries, even a lower number of laparoscopic hysterectomies were recorded, with the lowest figures in Croatia and Malta (9%) and Romania (3%) [9].

While analyzing these numbers, it is difficult to explain how laparoscopic cholecystectomy became a “gold standard” in benign gallbladder pathology in Romania, but the rest of the surgical procedures using minimally invasive techniques did not benefit from the same rate of development and implementation.

The lack of wide-spread techniques and familiarization with the laparoscopic instruments in benign hysterectomy can be easily noticed considering the presented numbers.

Literature studies have identified the following factors limiting the wide acceptance and implementation of laparoscopic hysterectomy: insufficient experience and training, lacking necessary equipment and support from colleagues [10].

**The objective** of this paper is to review the steps of the laparoscopic hysterectomy technique as well as to present the available variety of surgical instruments impartially so that the operative team can decide in an informed manner the model and characteristics of the equipment used.

### Surgical technique and laparoscopic instrumentation

#### Positioning the patient on the operating table

The lithotomy position, with the patient on her back and legs abducted, similar to gynecologic examining position but keeping an open-angle about 150 degrees between the thighs and body, as well as between the calves and thighs. During the intervention, the patient is placed in the Trendelenburg position in order to mobilize the small bowel loops from the pelvis and visualize the structures of the reproductive apparatus.

The use of pneumatic or foam mattress for leg and shoulder support has been shown to be associated with a low risk of peripheral neurological injury and low postoperative pain at this level [11]. Also, the use of the pneumatic mattress prevents the patient from sliding off the surgical table.

**Figure 1:**
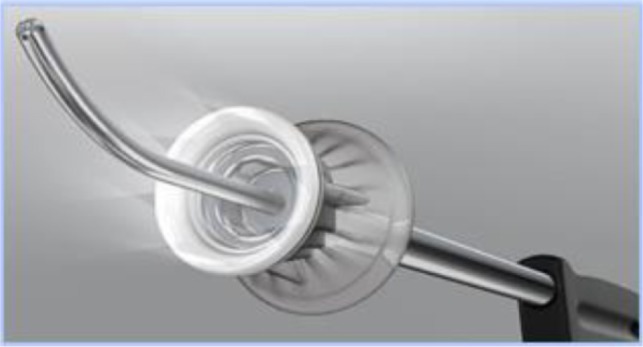


**Figure 2:**
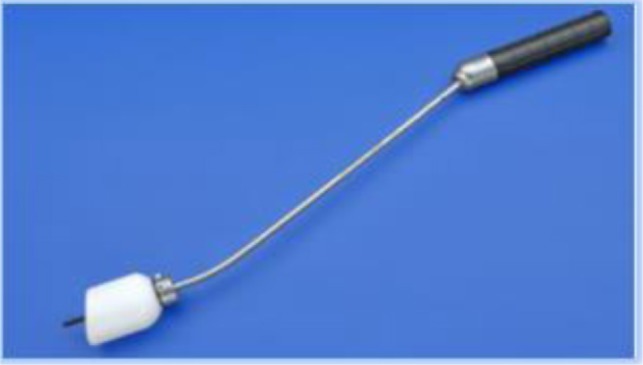


**Figure 3:**
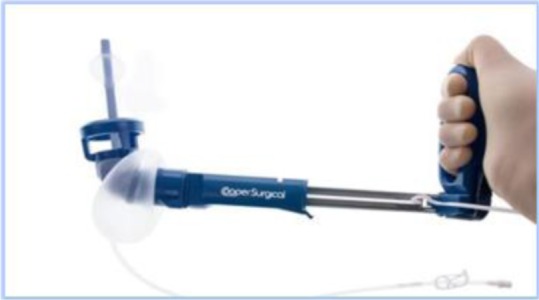


The technique continues with the introduction of the uterine manipulator, without which the operation cannot be completed. There are many available options:

a) SecuFix produced by Richard Wolf (fig. 1) which has a universal tip (adjustable for several dimensions, depending on the size of the cervix) that is illuminated, facilitating the visualization portion where the vagina surrounds the cervix.
b) a classic reusable manipulator like that proposed by Karl Storz (fig. 2): ceramic caps of various sizes and a metal handle that are simple to use;
c) Rumi II - golden standard in the United States (fig.3), has caps of various sizes that adapt to the diameter of the cervix, an intrauterine balloon that prevents accidental extraction of the manipulator during surgery, the shaft allowing a full 140 degrees of articulation at the cervix, facilitating a faster dissection of the uterus.

The use of any of the variants of uterine manipulators is the choice of the operating team, with the mention that the chosen one should have an adjustable cap that should be chosen according to the size of the cervix in order to avoid ureteral lesions (in the case of large caps) and difficult delimitation of the portion where the vagina surrounds the cervix (in the case of small caps).

**Figure 4:**
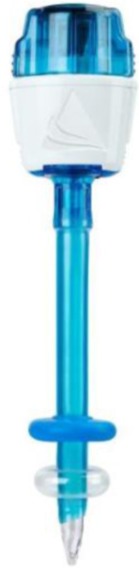
Applied Medical trocar

When performing pneumoperitoneum, using an open supraumbilical technique is preferred to blind access (the use of a Veress needle). The 10 mm trocar is inserted, and the gas is used for the insufflation of the abdominal cavity, typically 12 mm Hg. The trocar can be metallic, reusable, or disposable. If disposable trocars are available, it is recommended to use an optical trocar that is equipped with an inflatable balloon (Applied Medical), which prevents the accidental extraction of the trocar when handling the optical camera (fig.4)

Under visual control, the following trocars are introduced: two 5-mm trocars are placed bilaterally, laterally from the umbilicus, at the intersection between the spinoumbilical line with the midclavicular line, and another 5-mm trocar is positioned in the midline, 3-4 cm above the pubic symphysis. It can also be positioned in the right iliac fossa, laterally to the rectus abdominis muscle (fig. 5). Choosing the position of the fourth trocar depends on the operator’s will and experience; there are authors who believe that the latter variant is more ergonomic and more favorable when suturing the vagina [12].

**Figure 5:**
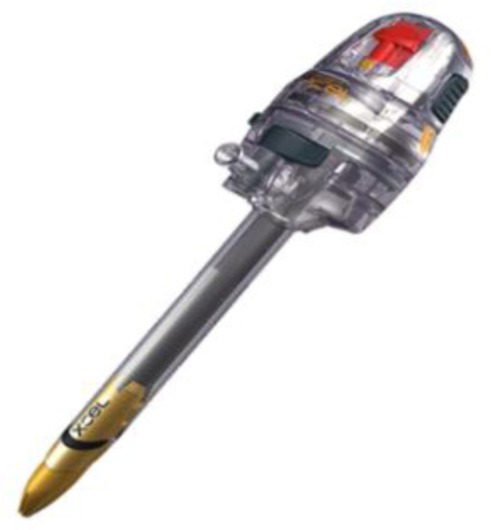
Ethicon trocar

If the uterus is very voluminous and cannot be extracted through the vagina, one of the 5-mm trocars placed laterally to the umbilicus will be replaced by a 12-mm trocar to allow the use of a morcellator, usually on the left side. Metallic, reusable or plastic trocars can be used.

Ethicon trocars are provided with a sliding blade at the tip, which helps transfascial penetration. (fig. 5) Their insertion should be done with extreme caution in order to avoid iatrogenic vascular or intestinal lesions. Medtronic trocars are represented by a plastic, less sharp, bladeless tip, which reduces the risk of injury at the time of insertion (fig. 6). The third category is represented by plastic trocars with balloon tips, which also prevent their accidental extraction during the operation (Applied Medical, fig. 4).

**Figure 5:**
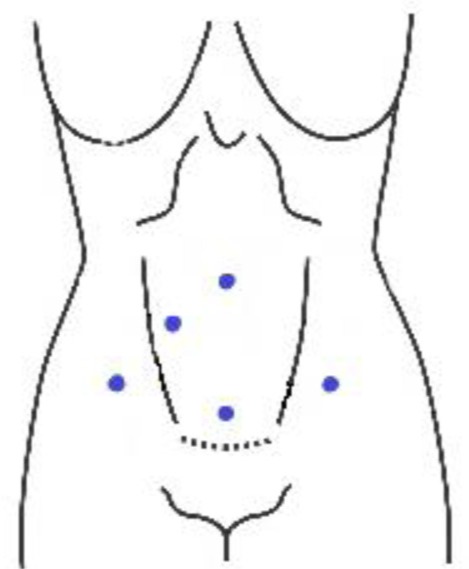
Anterior abdominal wall and trocar positioning in laparoscopic hysterectomy [13].

**Figure 6:**
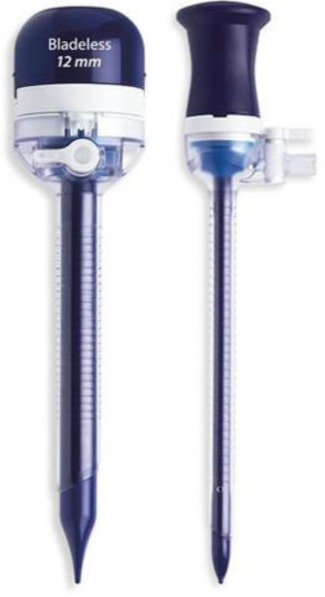
Medtronic trocars

**Image 1:**
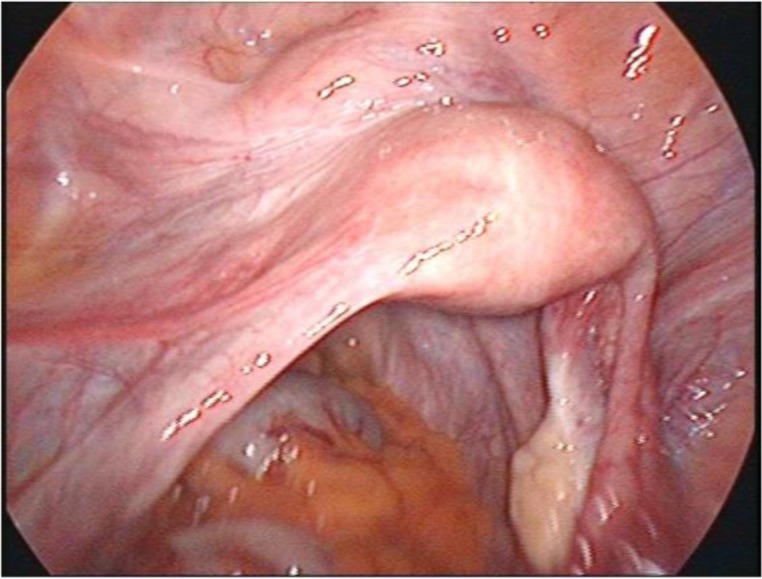
Manipulation of the uterus while identifying the key anatomical structures. Personal collection.

The surgical technique is largely standardized, being a mirror-like intervention: it can start on the right, and then the same steps are repeated on the left. The team member coordinating the uterine manipulator should push the uterus to the left of the patient so that the right flank is visible (Image 1), continuing in order to identify the following anatomical elements: the ovary and uterus, ureter, suspensory ligament of the ovary (infundibulopelvic ligament), round ligament and the vesico-uterine pouch.

**Figure 7:**
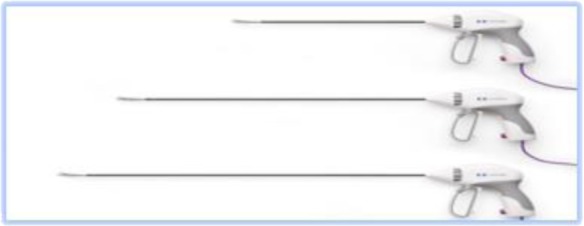


**Figure 8:**
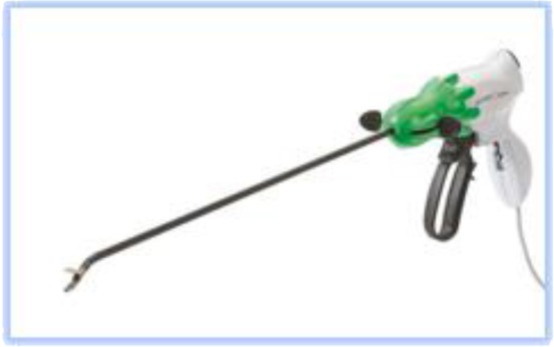


**Figure 9:**
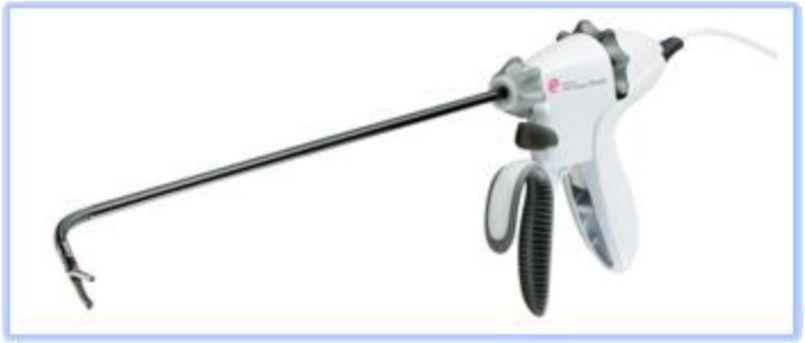


**Figure 10:**
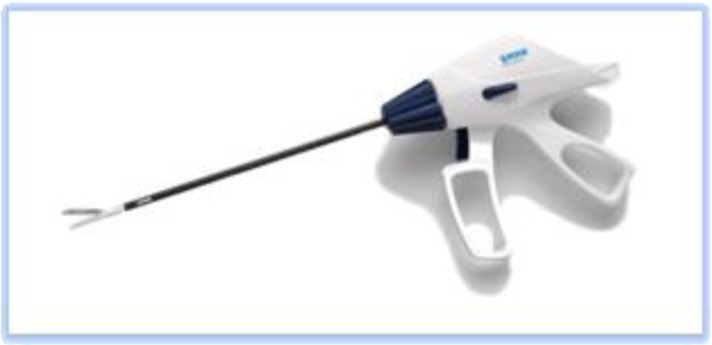


**Figure 11:**
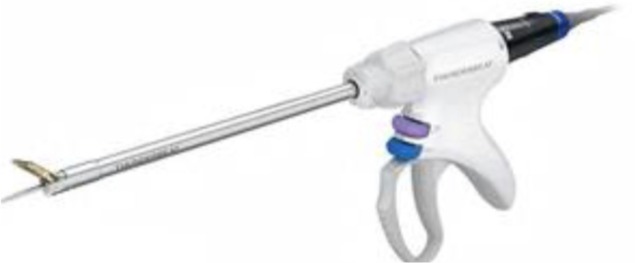


**Figure 12:**
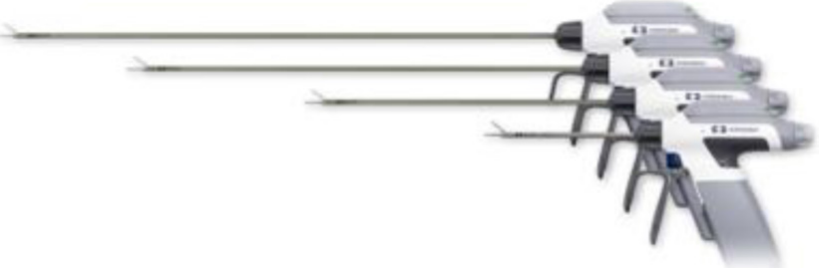


**Figure 13:**
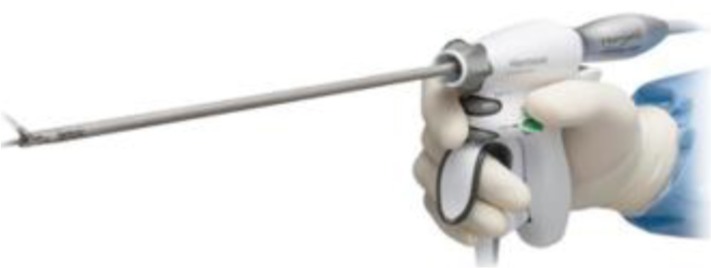


It also should be decided which kind of high energy (ultrasonic coagulation and cutting) device will be used. Two broad categories are available:

1. **Bipolar current** devices: LigaSure - Medtronic (fig. 7), Caiman - Aesculap (fig. 8), EnSeal -Ethicon (fig. 9) or BiCision - Erbe Elektromedizin (fig. 10).
2. **Radiofrequency devices**: Harmonic Ace 7 - Ethicon (Fig. 11), Sonicision Cordless Ultrasonic Dissection Device - Medtronic (Figure 12) or Thunderbeat - Olympus (Fig. 13)

**Figure 14:**
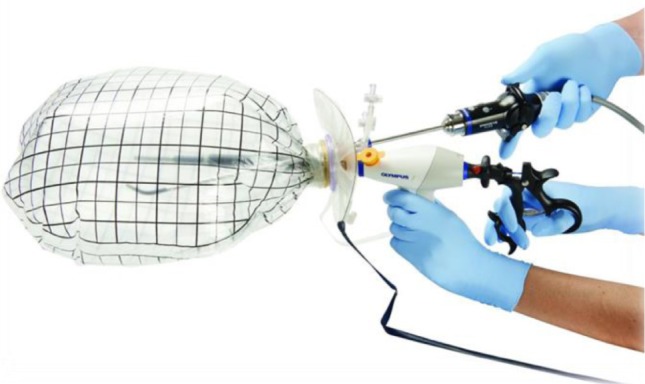
Endo-bag morcellator (Olympus)

Instruments in the first category can be used on vessels up to 7 mm in diameter with minimal Stergem postoperative bleeding risk. All these tools have similar features, and the operator`s option for one of the variants is a matter of preference and availability. Two of them (Caiman and EnSeal) have a flexible tip that can be oriented very easily, which is a significant feature, of great technical help. In the case of a larger uterus with difficult access to the deep pelvis and cervix, it is preferable to choose such a flexible, high-energy device.

The instruments in the second category can mostly be used on vessels up to 5 mm in diameter (additional attention should be paid to the uterine artery) and produce a combination of vapor and smoke during use, which can be disturbing in some situations. Stergem complet propozitia aceasta. Also, it should be noted that Sonicision is battery-operated and therefore portable and more ergonomic in use.

**Image 2:**
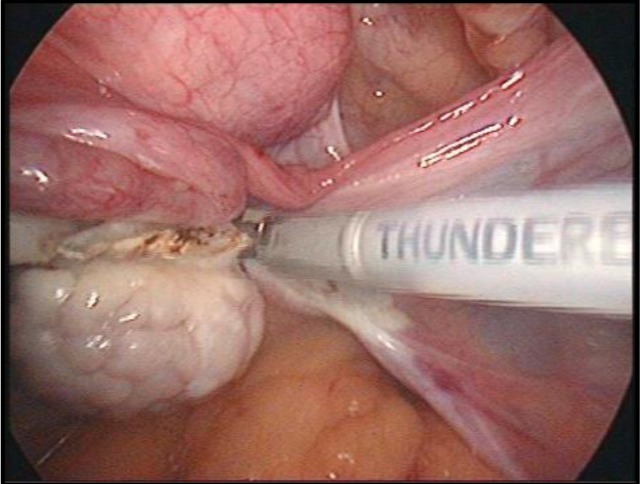
Coagulation of the infundibulopelvic ligament. Personal collection

**Image 3:**
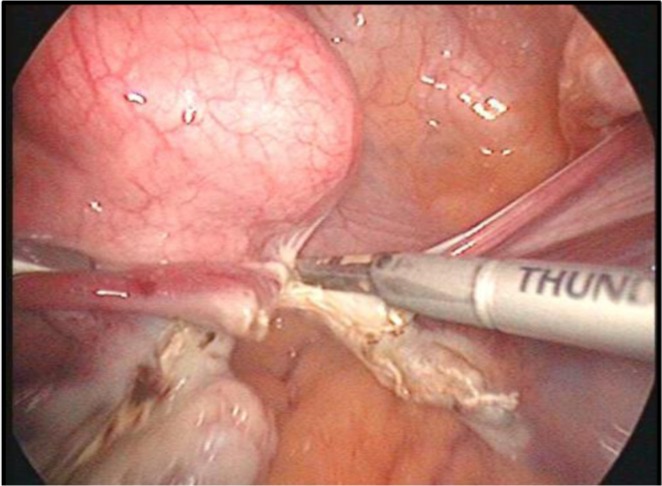
Dissection of the broad ligament. Personal collection

In the beginning, the utero-ovarian ligament (in case of simple hysterectomy) or the infundibulopelvic ligament (if salpingo-oophorectomy is associated) are identified, coagulated and cut (Image 2). For this step, as well as for the whole operation, either of the tools described in the two categories can be used. Once again, it is a matter of personal preference when choosing the devices used during the procedure, this paper aiming at an impartial presentation of the technological arsenal available at this time. The dissection advances with the coagulation and incision of the round ligament, the next step being to identify the space between the two layers of the broad ligament and advance caudally in this space, which, if correctly identified, should be avascular (Image 3).

**Image 4:**
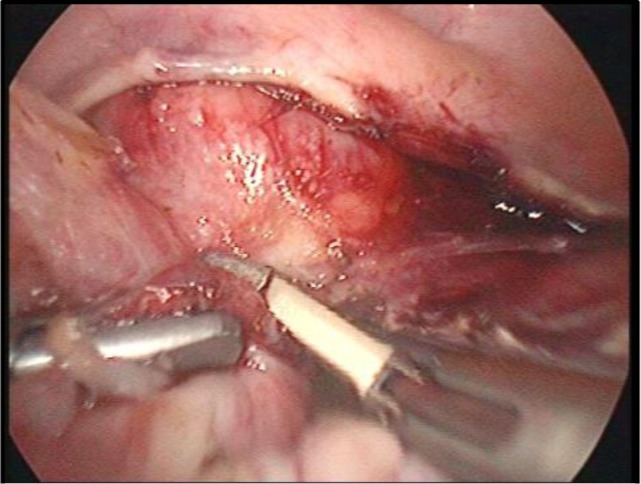
Coagulation of vascular elements on the right side. Personal collection

The anterior layer should be coagulated and dissected step by step, progressing caudally towards the uterine vessels located on the posterior sheet of the broad ligament. Rigorous identification is recommended; only after that, dissection and coagulation should be performed. Coagulation of the vascular elements using the high energy device is done at least 1 cm above the incision point of the pubocervical fascia. During this procedure, the role of the uterine manipulator is essential; it should be oriented to the contralateral side towards the flank in which the operation is performed (Image 4). Everything should be done as close as possible to the uterus, minimizing the risk of ureteral injury. The procedure continues in the vesico-uterine pouch; the dissection plane is angled between the urinary bladder and uterus, preventing thus bladder injury during peritoneal dissection. If this avascular plane, which guarantees dissection without bladder damage, is certainly not identified (sometimes because of adhesions following previous cesarean sections), it is recommended to cut the peritoneum with regular scissors since the possible occurrence of postoperative detachment of tissue which has undergone thermal injury and consequent uroperitoneum being demonstrated [14-16].

At this point of the operation, the dissection of the cervix continues tangent to it and advances at the same time in the ventral plane (between the urinary bladder and uterus). The dissection stops when it is estimated that the portion where the vagina surrounds the cervix has been reached. Now that the right side is completely dissected, the operation continues on the left side.

**Image 5:**
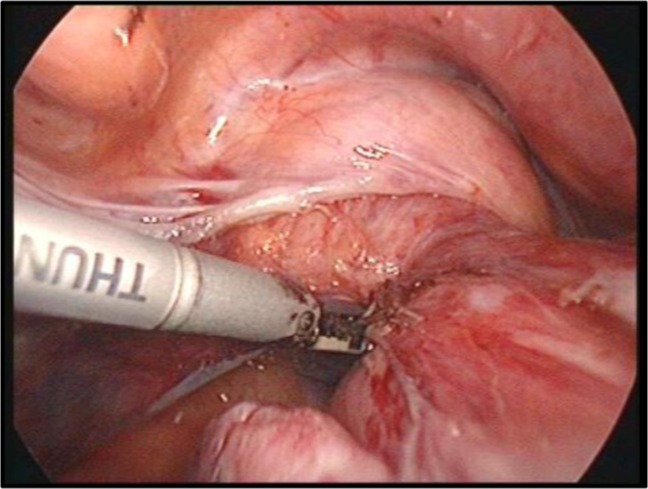
Coagulation of the vessels on the left side. Personal collection

**Image 6:**
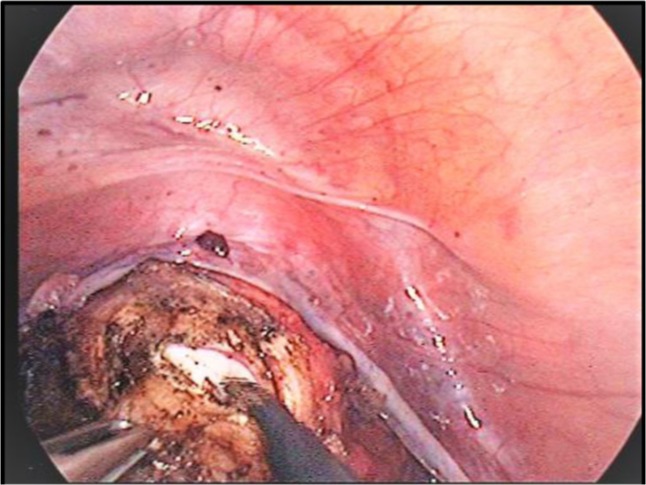
Sectioning of the vaginal wall. Personal collection

The surgeon’s assistant is asked to steer the uterine manipulator on the other side (in our example to the right of the patient) so that the left flank opens. Dissection via left flank approach is performed in the same manner (Image 5). At this point of the operation, the entire uterus and cervix are completely dissected, the entire structure being now attached only to the vaginal walls. With a monopolar hook or preferably with a monopolar spatula, the anterior vaginal wall is sectioned at maximum 1 cm from where the vagina surrounds the cervix (Image 6). The incision continues until the cap of the manipulator is viewed, making it easier to expose the vaginal insertion, minimizing the risk of injuries to other organs, such as the site where the ureter enters the bladder.

**Image 7:**
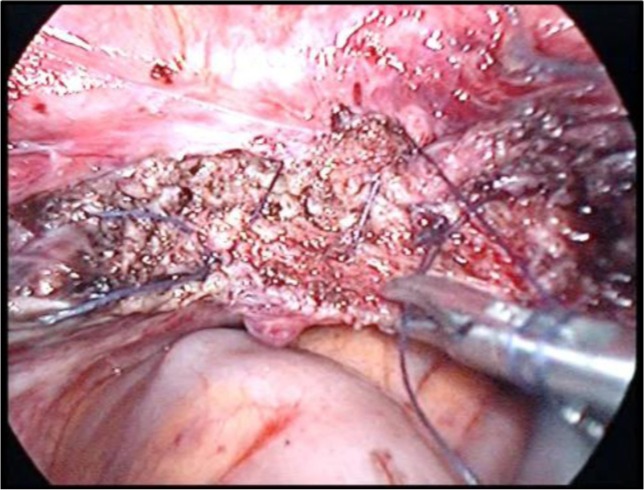
Suturing of the vaginal wall. Personal collection

The uterus can be extracted through the vagina or through a trans-parietal incision when larger myomas are being operated. Vaginal cuff hemostasis is then ensured, with the recommendation that coagulation should not occur extensively but only punctually, avoiding thus subsequent vaginal necrosis which usually is a source of important postoperative complications (abscesses, vaginal discharge, vaginal pain). Thereafter, the vagina is sutured intracorporeally using monofilament PDS (polydioxanone) (Image 7). The vaginal cuff may be suspended to the residual uterosacral ligaments.

Uterine morcellation can be tempted in large uteri but it is indicated to use endo-bags (because of oncological reasons) in order to prevent the accidental dissemination of malignant cells that may be present in an undiagnosed sarcoma. A morcellator is a tool that has a cylindrical cutting blade, designed to cut the uterine piece into small fragments inside the abdomen of the patient, which can then be extracted through the trocar of the instrument. It consists of a cylinder which inserts through the trocar and ends with a cutting blade in which the fibroma is inserted, shredded and then extracted by suction outside the abdominal cavity. The development of this instrument has arisen at the beginning of the 90´ as a result of the need for larger volume fibromas to be extracted through small incisions available in minimally invasive surgery [17-19]. Although, the FDA (Food and Drug Administration) has issued in 2014 a communication discouraging the use of power morcellation because of a reported case of a woman with fibroma who underwent intraabdominal morcellation, in which the histopathological exam revealed uterine sarcoma and the restaging surgery showed peritoneal metastasis [20]. Taking this into consideration, the European Society of Gynecological Endoscopy (ESGE) has organized a steering committee on fibroid morcellation in order to investigate and evaluate the risk of morcellation [21]. The risk of undiagnosed uterine sarcoma was reported in different studies, but most of them were retrospective or investigated only a few cases, between 0.49% and 0.056% [22-25]. The group concluded that there is not enough evidence to estimate the risk in individual patients, the complications of morcellation are rare and prospective data collection may clarify the issue on sarcoma risk in presumed fibroids and that technology of extracting tissue laparoscopically from the abdominal cavity should be perfected.

Safe endo-bag morcellation (in-bag morcellation) is investigated as it may possibly prevent morcellation complications. Preclinical and clinical results are promising but validating prospective studies are needed in order for in-bag morcellation to be the subject of a consensus recommendation [26-31].

## Conclusion

Analyzing the trend of laparoscopic hysterectomies in Europe, it can be noted that 22 of the 23 Member states of the European Union for which data are available from 2011-2016 reported an increased number of performed hysterectomies; the exception was Slovenia, which reported a decrease in frequency. Four countries reported that the number of laparoscopic hysterectomies doubled; three and four times more laparoscopic hysterectomies were performed in 2016 compared to 2011 in Romania and Hungary, respectively. Despite the increased number of laparoscopic hysterectomies performed between 2011 and 2016, Romania still ranks last in Europe (3%) [9]. The laparoscopic approach offers the advantages of minimal invasiveness: less pain, faster recovery, and early social reintegration; therefore, this trend of improvement should become more accepted.

## Conflict of Interest

The authors confirm that there are no conflicts of interest.
